# An unexpected turn of fortune: targeting TRAIL-Rs in KRAS-driven cancer

**DOI:** 10.1038/s41420-020-0249-4

**Published:** 2020-03-17

**Authors:** Silvia von Karstedt, Henning Walczak

**Affiliations:** 1 Department of Translational Genomics, Center of Integrated Oncology Cologne-Bonn, Medical Faculty, University of Cologne, Cologne, Germany; 2 CECAD Cluster of Excellence, University of Cologne, Cologne, Germany; 3 Center for Molecular Medicine Cologne, Medical Faculty, University Hospital of Cologne, Cologne, Germany; 4 Institute for Biochemistry I, Medical Faculty, University of Cologne, Cologne, Germany; 5 Centre for Cell Death, Cancer, and Inflammation (CCCI), UCL Cancer Institute, University College, London, WC1E 6BT UK

**Keywords:** Cell death, Cell biology

## Abstract

Twenty-one percent of all human cancers bear constitutively activating mutations in the proto-oncogene *KRAS*. This incidence is substantially higher in some of the most inherently therapy-resistant cancers including 30% of non-small cell lung cancers (NSCLC), 50% of colorectal cancers, and 95% of pancreatic ductal adenocarcinomas (PDAC). Importantly, survival of patients with KRAS-mutated PDAC and NSCLC has not significantly improved since the 1970s highlighting an urgent need to re-examine how oncogenic KRAS influences cell death signaling outputs. Interestingly, cancers expressing oncogenic KRAS manage to escape antitumor immunity via upregulation of programmed cell death 1 ligand 1 (PD-L1). Recently, the development of next-generation KRAS^G12C^-selective inhibitors has shown therapeutic efficacy by triggering antitumor immunity. Yet, clinical trials testing immune checkpoint blockade in KRAS-mutated cancers have yielded disappointing results suggesting other, additional means endow these tumors with the capacity to escape immune recognition. Intriguingly, oncogenic KRAS reprograms regulated cell death pathways triggered by death receptors of the tumor necrosis factor (TNF) receptor superfamily. Perverting the course of their intended function, KRAS-mutated cancers use endogenous TNF-related apoptosis-inducing ligand (TRAIL) and its receptor(s) to promote tumor growth and metastases. Yet, endogenous TRAIL–TRAIL-receptor signaling can be therapeutically targeted and, excitingly, this may not only counteract oncogenic KRAS-driven cancer cell migration, invasion, and metastasis, but also the immunosuppressive reprogramming of the tumor microenvironment it causes. Here, we provide a concise summary of the current literature on oncogenic KRAS-mediated reprogramming of cell death signaling and antitumor immunity with the aim to open novel perspectives on combinatorial treatment strategies involving death receptor targeting.

## Facts

- KRAS is the most frequently mutated proto-oncogene in human cancers.
- KRAS-mutated cancers show poor checkpoint blockade response.
- TNF superfamily ligand signaling is re-programmed to mediate migration, invasion, and metastasis in KRAS-mutated cancer.

## Open questions

- Can antagonistic targeting of TNF superfamily ligand signaling impact KRAS-driven cancer?
- How does targeting of TNF superfamily ligand signaling influence the tumor immune environment?
- Could targeting of TNF superfamily ligand signaling serve as an alternative immunotherapeutic approach for KRAS-mutated cancers?

## KRAS-mutated cancers: cancers with unmet needs

Survival rates for most KRAS-mutated cancers such as non-small cell lung cancer (NSCLC) and pancreatic ductal adenocarcinoma (PDAC) have not significantly improved since the 1970s. This dire situation is, in part, caused by the fact that KRAS-mutant cancers including PDAC are commonly only diagnosed at an advanced stage due to the absence of early symptoms. Whereas many KRAS effector pathways have been targeted using small molecule inhibitors against kinases activated downstream of KRAS^1^, their clinical application has raised concern over toxicity and even paradoxical effects promoting the disease through transactivation of wild-type (WT) v-raf murine sarcoma viral oncogene homolog B1(BRAF)^2^. Other potentially KRAS-mutant-selective therapeutic targets have emerged from synthetic lethality screens comparing effects on KRAS-mutated vs. KRAS WT cell lines^3–7^. However, the fact that activating point mutations uniquely occur in tumor cells has fueled efforts to develop small molecule inhibitors designed to selectively target these point mutant variants. As such, inhibitors that selectively bind and inactivate KRAS^G12C^ ^8,9^ have been developed showing promising results in first human trials^10^. Whilst hopes are high that this may provide a silver bullet against KRAS^G12C^-mutated cancers, the fate of inhibitors of oncogenic BRAF^V600E^ has taught us to be careful with such hopes. Most importantly, however, cancers driven by all other forms of point-mutated *KRAS* including KRAS^G12D^, the most prevalent KRAS point mutation across all human cancers^11^, remain difficult to treat. Apart from these drug discovery-driven approaches, cell biological research has unearthed several important concepts highlighting mechanisms of how oncogenic KRAS manipulates antitumor immunity and physiological cell death signaling. These two concepts, which turn out to be a lot more closely interlinked at the level of immune cell–tumor cell encounters than previously thought, will be discussed in this review.

## Patients with KRAS-mutated PDAC do not benefit from immune checkpoint blockade

Undeniably, the second decade of the third millennium has been the decade in which the inhibition of so-called immune checkpoints which serve to prevent autoimmunity^12^ has come of age in cancer therapy. The two immune checkpoint receptor–ligand systems whose inhibition has proven to be successful in the cancer clinic are the cytotoxic T lymphocyte-associated protein 4 (CTLA4)–B7-1/B7-2^13^ and the programmed cell death protein-1 (PD-1)–programmed cell death 1 ligand 1 (PD-L1) systems^14^. Indeed, immune checkpoint blockade (ICB) has become a gamechanger in the therapy of certain cancers, especially advanced melanoma, with unprecedented response rates^15–18^. Interestingly, expression of oncogenic RAS has been shown to upregulate PD-L1 via stabilization of its mRNA^19^, suggesting this as a possible means by which KRAS-mutated cancers may escape immunosurveillance. However, in PDAC, ICB so far failed to provide clinical benefit^20–22^ (summarized in Table 1, also reviewed in^23,24^). This may, in part, be caused by the fact that cancers require a high tumor mutational burden (TMB) to achieve therapeutic efficacy via checkpoint blockade^25,26^. PDAC, the cancer with the highest incidence of KRAS mutations, however, does not present with very high TMB^27^. In addition, tumors that initially respond to ICB due to high TMB may acquire resistance by inactivation of the IFN-γ pathway^28,29^. Intriguingly, however, an inhibitor which selectively targets oncogenic KRAS^G12C^, AMG510 was recently found to enable effective antitumor immunity^10^. Interestingly, this immune response was sufficient to also attack KRAS^G12D^-expressing tumors in trans demonstrating that antitumor immunity is prevented by oncogenic KRAS, but can be re-instated through its inhibition. Together with the fact that PDAC patients poorly respond to ICB, these results imply that an alternative mechanism functionally similar to, but molecularly distinct from, conventional immune checkpoints is responsible for oncogenic KRAS-driven reprogramming of the tumor immune microenvironment (TIME) and, consequently, for immune evasion of KRAS-mutated cancers. Another possibility is that, besides conventional immune checkpoints, this currently elusive immune checkpoint provides an additional layer of protection preventing recognition and destruction by the immune system.

**Table 1:** Selection of published and ongoing clinical trials testing immune checkpoint blockade (ICB) in pancreatic cancer.

Trial identifier	Cancer types	Treatment groups	Response	Reference
Completed	PDAC, NSCLC, Melanoma, CRC, renal cancer, ovarian cancer, gastric cancer, breast cancer	BMS-936559 (anti-PD-L1)	0% (PDAC)	^21^
Completed	Previously treated PDAC	Ipilimumab	0%, 1 delayed PR	^22^
Completed	Previously treated PDAC	Durvalumab (D; anti-PD-L1) vs. Durvalumab + Tremelimumab (T; anti-CTLA-4)	0% 3% (D/T) PR	^20^
NCT03871959	Advanced/meta-static PDAC/CRC	Pembrolizumab (Pe; anti-PD-1) vs. Pembrolizumab + Debio1143 (Db; SMAC mimetic)	pending	N/A
NCT03634332	Previously treated PDAC	Pembrolizumab (Pe; anti-PD-1) vs. Pembrolizumab + PEGPH20 (hyaluronidase)	pending	N/A
NCT03767582	Previously treated PDAC	Nivolumab (anti-PD-1) combination with BMS-813160 (CCR2/CCR5 Dual Antagonist) and GVAX	pending	N/A
NCT02777710	Advanced/meta-static PDAC/CRC	Durvalumab (D; anti-PD-L1) vs. Durvalumab + Pexidartinib (P; kinase inhibitor against colony-stimulating factor-1 receptor; CSF1R)	pending	N/A
NCT03727880	Resectable PDAC	Pembrolizumab (Pe; anti-PD-1) vs. Pembrolizumab + Defactinib (De; inhibitor against focal adhesion kinase, FAK)	pending	N/A

*PR* partial response, *NSCLC* non-small cell lung cancer, *CRC* colorectal cancer.

## The TNF superfamily member TRAIL and its receptors

Tumor necrosis factor is the founding member of the TNF superfamily (TNF-SF) of cytokines. Members of the TNF-SF are synthesized as type II transmembrane proteins. They are frequently expressed by activated immune cells but can also be cleaved from their surface and can then act as soluble cytokines^30^. Tumors are constitutively exposed to the functional consequences of signaling induced by various members of the TNF-SF, produced by diverse immune cells in the TIME, as part of the immune cell-mediated antitumor attack. Members of the TNF-SF bind to specific receptors that form part of a corresponding protein family, the TNF-receptor (TNFR)-SF. Members of this family are characterized by the presence of up to six repeats of a characteristic cysteine-rich domain within their extracellular portion. Six members of the TNFR-SF contain a so-called intracellular death domain (DD), which is required for cell death induction. These receptors are therefore also referred to as death receptors. The most intensively studied receptors of this type are TNF-receptor 1 (TNF-R1), CD95 (Fas/APO-1), and the two TRAIL death receptors, TRAIL-receptor 1 and 2 (TRAIL-R1 and TRAIL-R2)^31^.

Within this protein family, TRAIL^32^ received a high level of interest due to its capacity to selectively kill tumor cells, importantly without killing any essential normal cell type^33,34^. TRAIL has been shown to bind five different cellular receptors, which can be subdivided into the ones that contain a DD, TRAIL-R1 (DR4), and TRAIL-R2 (DR5), and those that do not, TRAIL-R3, TRAIL-R4, and osteoprotegerin (OPG) (reviewed in^35–37^). OPG is a soluble regulatory receptor for RANKL but has been shown to also bind TRAIL. Mice only express one DD-containing receptor for TRAIL (mTRAIL-R/MK) which is equally homologous to human TRAIL-R1 and TRAIL-R2. Moreover, two additional mouse receptors, mDcTRAIL-R1 and mDcTRAIL-R2, have been described which lack an intracellular DD. All of the alternative human and murine TRAIL-Rs have been suggested to function as ligand “decoys”, competing for TRAIL binding to the cell death-inducing receptors, a theoretical concept which has been rather difficult to validate under non-overexpression conditions. Recently, the membrane-proximal domain (MPD) within human TRAIL-R2, an amino acid sequence which is shared with mTRAIL-R, was identified to be required for Rac1 activation and migration in KRAS-driven cancers^38^. Of note, the MPD of TRAIL-R2 shares a lower degree of conservation with that of TRAIL-R1 than with the MPD of CD95, a death receptor which has been shown to activate Rac1 in neurons^39^.

## TRAIL-induced signaling pathways: a brief recapitulation of the corner stones

### FADD/Caspase 8-dependent apoptotic TRAIL signaling

Upon binding of TRAIL, TRAIL-R1 and TRAIL-R2 form homotrimeric, possibly also heterotrimeric receptor–ligand complexes that are assembled into large, higher-order complexes^40,41^. Receptor oligomerization allows for the recruitment of Fas-associated protein with a death domain (FADD) via its DD to the DD of TRAIL-R1 and/or TRAIL-R2. Making use of its death-effector domain (DED), FADD recruits the initiator caspases 8 and 10 via their respective DEDs. The resulting complex is referred to as the death-inducing signaling complex (DISC)^42–44^. Upon activation and cleavage, active caspases 8 and 10 are released from the DISC allowing for the cleavage of cytosolic substrates including effector caspases such as caspase 3. Finally, effector caspases cleave inhibitor of caspase-activated DNAse (iCAD) leading to the activation of caspase-activated DNAse (CAD), which is responsible for the hallmark DNA fragmentation observed during apoptosis^45^. In so-called type I cells, DISC formation is sufficient to induce extrinsic apoptosis in target cells. Yet in type II cells, DISC formation alone is insufficient for full activation of effector caspases due to the inhibitory effect exerted by the X-linked inhibitor of apoptosis protein (XIAP) ^46^. However, caspase 8 also cleaves Bid. Truncated Bid aids Bax- and Bak-mediated mitochondrial outer membrane permeabilization (MOMP)^47^. MOMP leads to the release of Smac/DIABLO into the cytosol, which antagonizes XIAP. In type II but not in type I cells, this part of the pathway is required for cell death induction, providing a molecular explanation for the type I–type II cell dichotomy. Another consequence of MOMP is the release of cytochrome C from mitochondria which, together with Apaf1 and the initiator caspase 9, forms the apoptosome, a multimeric caspase-activatory complex which is also capable of activating effector caspases^48^—essentially serving as an “intracellular DISC”.

### RIPK1/RIPK3-dependent necroptotic TRAIL signaling

Apoptotic DISC components have been shown to actively suppress aberrant induction of necroptosis, a type of regulated or programmed necrosis^49^. This was first proven genetically by showing that embryonic lethality of deficiency in caspase 8 or FADD could be reversed by co-ablation of receptor-interacting serine/threonine-protein kinase 3 (RIPK3)^50,51^ or RIPK1^52,53^. Therefore, unlike apoptosis, necroptosis is not mediated but suppressed by caspase activity. Instead, necroptosis is executed by the kinase activity of RIPK1 and RIPK3^54^. Whilst most studies on necroptosis have been in the context of TNF stimulation, CD95L and TRAIL can be necroptotic under certain conditions. Interestingly, the linear ubiquitin chain assembly complex (LUBAC) forms part of both, the TRAIL-R1/2-associated complex I and the cytoplasmic complex II^55^ of TRAIL signaling, limiting TRAIL-induced apoptosis and necroptosis^56^.

### RIPK1-dependent pro-tumor TRAIL signaling

Signaling via TRAIL-R1, TRAIL-R2 and TRAIL-R4 can induce activation of nuclear factor kappa-light-chain-enhancer of activated B cells (NF-κB), a master regulator of the immune response. In the context of TRAIL stimulation, NF-κB promotes migration and invasion^57^. Interestingly, RIPK1 presence in the native TRAIL DISC was shown to augment NF-κB activation when caspases are inhibited^58^. Moreover, TRAIL-induced NF-kB activation is increased by FADD presence^59^, caspase inhibition, and LUBAC activity^56^. Apart from a role for RIPK1 in the DISC as an activator of NF-κB, the formation of a secondary intracellular signaling complex has been proposed to activate NF-κB, mitogen-activated protein kinases (MAPKs), JNK, and p38 pathways^55^. In vitro, TRAIL can trigger migration in a RIPK1-, Schmidt-Ruppin A-2 viral oncogene homolog (Src)- and Signal transducer and activator of transcription 3 (STAT3)-dependent manner^60^. Lastly, RIPK1 has been reported to be required for TRAIL-induced NF-κB activation^61^, survival, and proliferation.

## Oncogenic KRAS-mediated rewiring of TRAIL signaling

Activating point mutations in the small GTPase KRAS lead to its constitutive activation^1^. Thereby, cancers with KRAS mutations experience constitutively elevated signaling via KRAS effector pathways such as activation of the phosphatidylinositol 3 kinase (PI3) kinase pathway via direct interaction of KRAS with the catalytic PI3K subunit p110α^62^. Interestingly, p110α overexpression in melanocytes can protect cells from TRAIL-induced apoptosis^63^. Moreover, small molecule inhibition of PI3K was shown to sensitize to TRAIL-induced apoptosis through elevating Bid expression^64^. Yet, studies using small molecules inhibitors for PI3K without further target validation have to be taken with a note of caution as the kinase inhibitor PIK-75, thought to act through inhibition of p110α, sensitizes cancer cells to TRAIL-induced apoptosis by inhibiting CDK9, whereas specific inhibitors of p110α failed to exert this effect^65^.

**Fig. 1:**
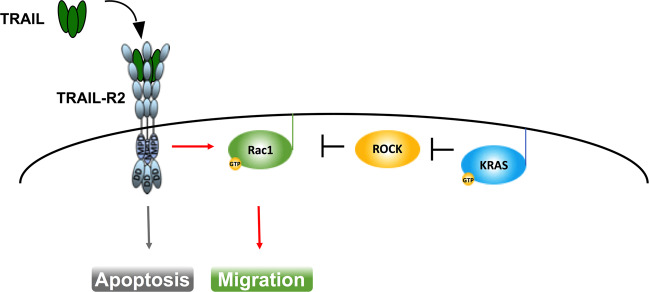
KRAS-mediated rewiring of TRAIL signaling. Cancer cell-produced TRAIL can bin TRAIL-R2 leading to autocrine cell-autonomous stimulation of Rac1-dependent migration. This signal is independent of the death domain (DD) but requires the membrane-proximal domain (MPD) of TRAIL-R2. Rac1 activity is normally inhibited by ROCK. Upon oncogenic activation of KRAS, KRAS inhibits ROCK, thereby releasing Rac1 to be fully activated by TRAIL-R2.

Another major effector pathway activated by oncogenic KRAS is the MAPK-family member extracellular regulated kinase (ERK), which can also be activated by TRAIL^66^. Importantly, inactivation of ERK signaling as a consequence of cellular detachment can sensitize cancer cells to TRAIL-induced apoptosis^67^. Given these and other studies, it was expected that KRAS-mutated cells would be more resistant to TRAIL-induced apoptosis. Yet, it came as a surprise when oncogenic KRAS was found to not only render colorectal cancer cells resistant to TRAIL and CD95L, but to convert the respective ligand-induced signals into migration-activating ones^68^. Moreover, treatment with exogenous TRAIL promoted KRAS-mutated PDAC metastases^69^. Whilst these studies highlighted KRAS mutations as contraindication for TRAIL-R agonistic treatments, the function and reasons for high endogenous expression of TRAIL and TRAIL-Rs observed in these cancers remained unknown. Intriguingly, however, these findings proposed an intriguing hypothesis: cancers might profit from highly expressing TRAIL and TRAIL-Rs. Indeed, this hypothesis proved to be correct as endogenous tumor cell-expressed mTRAIL-R promoted KRAS-driven NSCLC and PDAC growth and metastasis by activating the small GTPase Rac1 in vivo^38^. Moreover, Rho-associated protein kinase (ROCK) inhibition by oncogenic KRAS was sufficient to enable endogenous TRAIL-R2-mediated migration also in KRAS wild-type cells^38^ (concept summarized in Fig. 1). The latter observation implies that this previously unrecognized mechanism may be more widely utilized, also in oncogenic contexts beyond cancer with mutated KRAS. Of note, c-Raf which can function as a KRAS effector upstream of ERK was shown to suppress ROCK, thereby regulating CD95-mediated cell death^70^. Hence, inhibition of the interactions of endogenous TRAIL–TRAIL-R2 and/or CD95L–CD95 may prove a viable therapeutic concept in oncogene-activated cancers with ROCK inhibition.

## Immune-modulatory activities of TRAIL–TRAIL-R interaction: an immune checkpoint for KRAS-mutated cancer?

The therapeutic effect of inhibition of TRAIL or CD95L may, however, extend beyond the reach of blocking their role in tumor biology. In this context, it appears particularly noteworthy that mice deficient for TRAIL-R^71^ or TRAIL are more susceptible to induction of autoimmune diabetes^72^ and arthritis^73^ suggesting a function for the TRAIL–TRAIL-R system in preventing autoimmunity. In line with this concept, TRAIL expression on T cells was shown to induce tolerance upon encounter with potentially immunogenic antigens in order to prevent aberrant immune responses^74^. Moreover, TRAIL upregulation on CD4+ Foxp3+ regulatory T cells (Tregs) was responsible for the elimination of T effector cells in a skin graft model and, thereby, for suppression of anti-graft immunity^75^. In addition, co-activation of TRAIL-Rs and the T-cell receptor has been shown to suppress T-cell activation^76^.

**Fig. 2:**
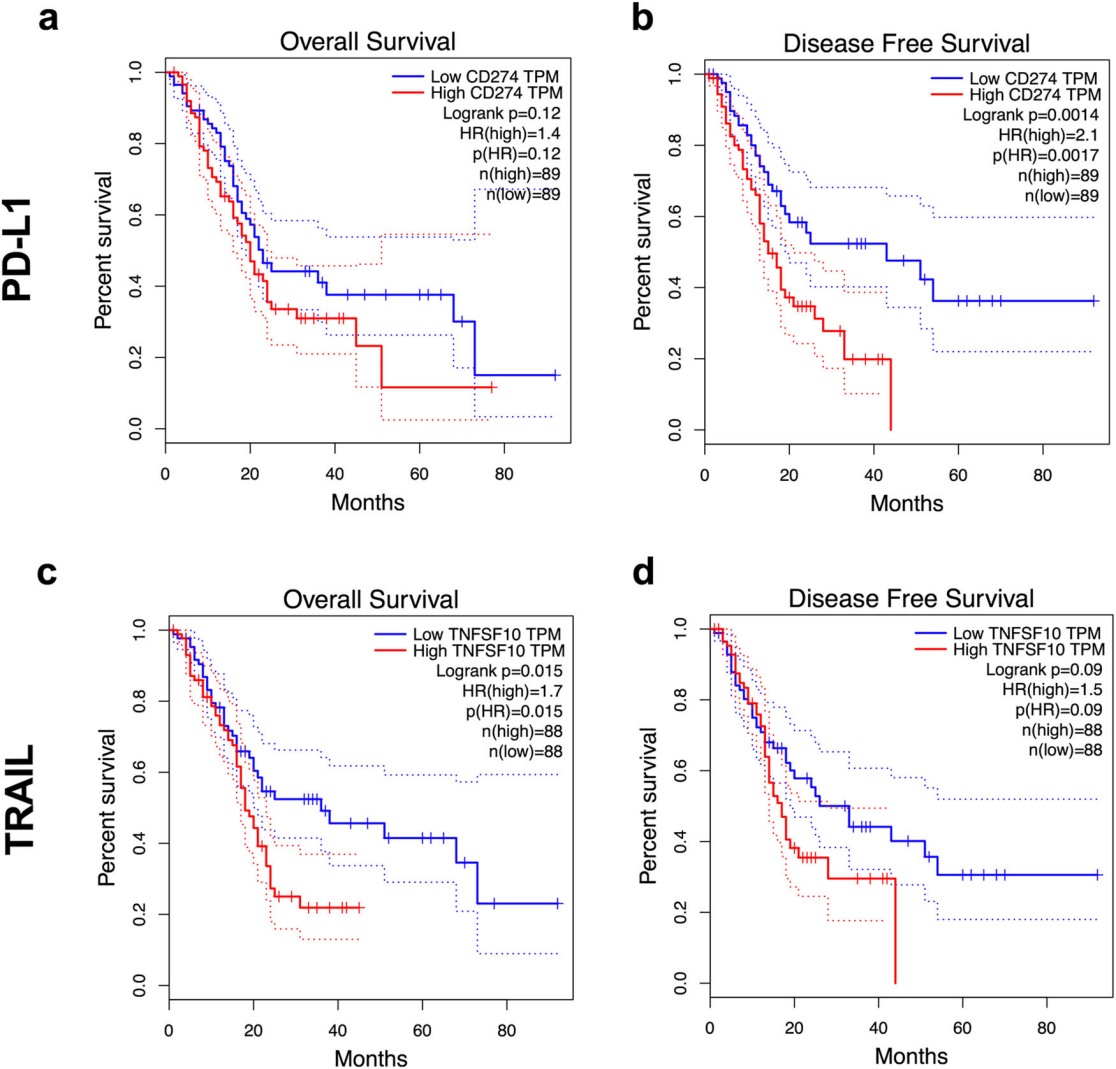
TRAIL expression is a superior marker of overall survival in PDAC. **a**, **c** The TCGA PDAC dataset (PAAD) was analyzed for overall survival split by median expression of PD-L1 (CD274, **a**) or TRAIL (TNFSF10, **c**). **b**, **d** Data as in **a**, **c** were analyzed for disease free survival. Kaplan-Meier survival plots are shown. The GEPIA analysis tool was used (http://gepia.cancer-pku.cn).

**Fig. 3:**
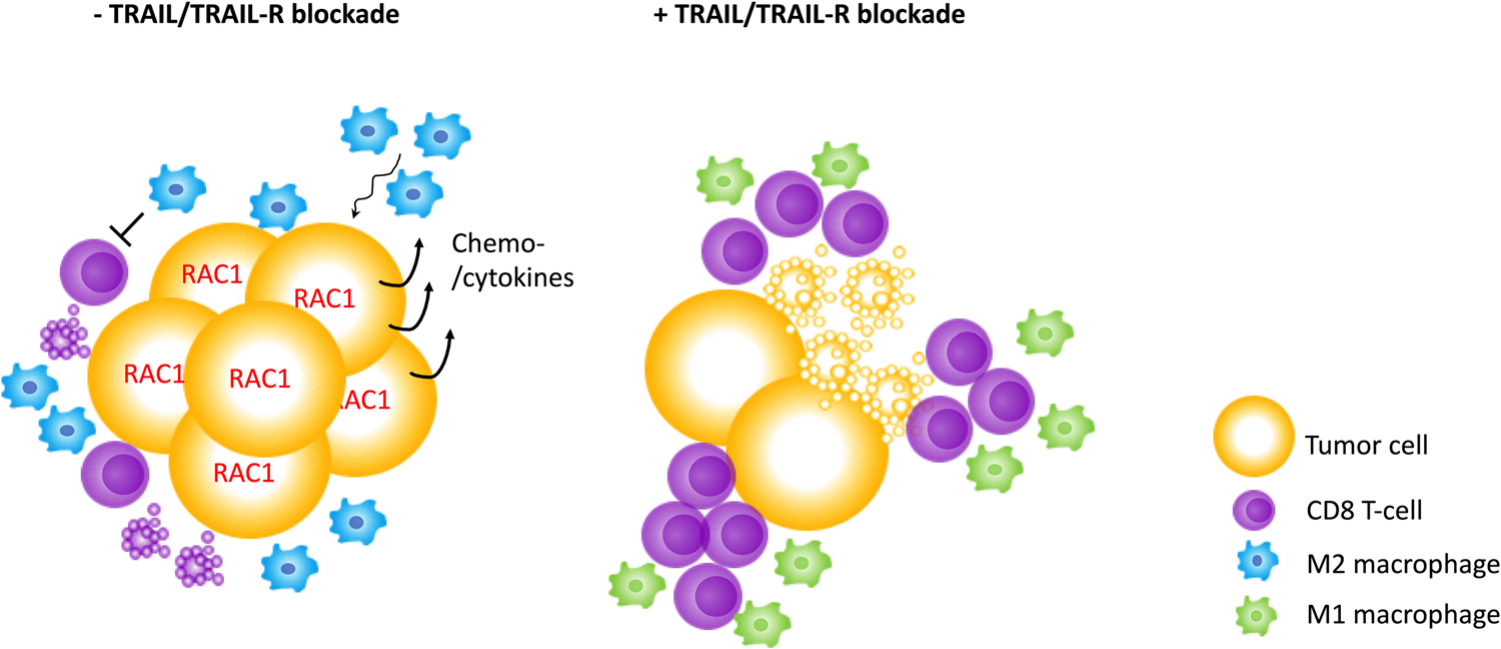
Potential impact of TRAIL/TRAIL-R blockade on tumor cell intrinsic signaling and reprograming of the tumor immune microenvironment (TIME). Endogenous TRAIL/TRAIL-R signaling eliminates activated CD8 T cells and promotes Rac1 activation and M2-polarizing chemo-/cytokine induction in tumor cells. Blockade of TRAIL/TRAIL-R expression prolongs survival of activated CD8 T cells, allows for an M1 polarized TIME and abrogates tumor cell Rac1 activity.

Intriguingly, many biological effects observed for endogenous TRAIL–TRAIL-R signaling are reminiscent of the effects observed for immune checkpoints preventing autoimmune disease^12^. In a similar manner to the PD-1–PD-L1 system, expression of TRAIL and its receptors is frequently upregulated in cancer^38,77–79^. Interestingly, in PDAC, a cancer in which conventional ICB failed to provide clinical benefit, high PD-L1 expression only correlates with shortened progression-free but not overall survival (Fig. 2a, b). Importantly, in the same PDAC patient cohort, high TRAIL expression does not correlate with progression-free but with shortened overall survival (Fig. 2c, d), suggesting that the inhibition of TRAIL may provide therapeutic benefit for PDAC patients. Endogenous TRAIL-R2 was shown to promote KRAS-driven cancer through cancer cell-autonomous Rac1 activation and non-cancer-cell-autonomous reprogramming of the TIME into one conducive of type 2 macrophage accumulation^38,59^. Thus, blocking the TRAIL–TRAIL-R2 interaction may be beneficial for patients with KRAS-mutated cancers by acting on three levels: (i) by blocking cell-autonomous Rac1 activation; (ii) through inhibiting the creation of a type 2 macrophage-conducive TIME; and (iii) by prolonging CD8 T-cell activation, allowing for an increased level of “auto-reactive” immunity functionally similar to, but, importantly, molecularly distinct from, conventional ICB (concepts summarized in Fig. 3).

## Conclusions

Although the discovery of conventional ICB has revolutionized the therapy of several cancers, unfortunately PDAC, the cancer with the highest incidence of KRAS mutations, does not form part of this group. Reasons for this may partly lie in poor immunogenicity and poor perfusion. Yet, KRAS-mutated tumors appear to drive immune evasion via alternative means, in addition to manipulating PD-L1 expression. Interestingly, KRAS-mutated cancers often highly express TRAIL and TRAIL-Rs. Whereas KRAS-mutated tumors are more resistant to induction of apoptosis by ectopically added TRAIL, they engage the endogenous TRAIL–TRAIL-R system in disease progression. In addition, the survival of activated CD8 T cells is regulated via TRAIL–TRAIL-R. We conclude that inhibiting endogenous TRAIL in KRAS-mutated cancers may not only inhibit tumor growth, invasion, and metastasis, but also enhance adaptive immunity against these cancers. In keeping with the irony of PD-1, being originally named for its supposed function as an inducer of programmed death, the bona fide TRAIL–TRAIL-R and CD95L–CD95 death receptor–ligand systems may turn out to serve as alternative, possibly additional immune checkpoint systems whose inhibition may prove essential to extend the ICB concept beyond the conventional ICBs, importantly, also to KRAS-mutated cancer.
